# Combination Drug Therapy for the Management of Alzheimer’s Disease

**DOI:** 10.3390/ijms21093272

**Published:** 2020-05-05

**Authors:** Md. Tanvir Kabir, Md. Sahab Uddin, Abdullah Al Mamun, Philippe Jeandet, Lotfi Aleya, Rasha A. Mansouri, Ghulam Md Ashraf, Bijo Mathew, May N. Bin-Jumah, Mohamed M. Abdel-Daim

**Affiliations:** 1 Department of Pharmacy, BRAC University, Dhaka 1212, Bangladesh; tanvir_kbr@yahoo.com; 2 Department of Pharmacy, Southeast University, Dhaka 1213, Bangladesh; aam.pharma@hotmail.com; 3 Pharmakon Neuroscience Research Network, Dhaka 1207, Bangladesh; 4 Research Unit, Induced Resistance and Plant Bioprotection, EA 4707, SFR Condorcet FR CNRS 3417, Faculty of Sciences, University of Reims Champagne-Ardenne, P.O. Box 1039, 51687 Reims CEDEX 2, France; philippe.jeandet@univ-reims.fr; 5 Chrono-Environnement Laboratory, UMR CNRS 6249, Bourgogne Franche-Comté University, F-25030 Besançon, France; lotfi.aleya@univ-fcomte.fr; 6 Department of Biochemistry, Faculty of Sciences, King Abdulaziz University, Jeddah 21589, Saudi Arabia; ramansouri@gmail.com; 7 King Fahd Medical Research Center, King Abdulaziz University, Jeddah 21589, Saudi Arabia; ashraf.gm@gmail.com; 8 Department of Medical Laboratory Technology, Faculty of Applied Medical Sciences, King Abdulaziz University, Jeddah 21589, Saudi Arabia; 9 Division of Drug Design and Medicinal Chemistry Research Lab, Department of Pharmaceutical Chemistry, Ahalia School of Pharmacy, Palakkad 678557, India; bijovilaventgu@gmail.com; 10 Department of Biology, College of Science, Princess Nourah bint Abdulrahman University, Riyadh 11474, Saudi Arabia; may_binjumah@outlook.com; 11 Department of Zoology, College of Science, King Saud University, P.O. Box 2455, Riyadh 11451, Saudi Arabia; abdeldaim.m@vet.suez.edu.eg; 12 Pharmacology Department, Faculty of Veterinary Medicine, Suez Canal University, Ismailia 41522, Egypt

**Keywords:** combination therapy, dementia, Alzheimer’s disease, multi-target-directed ligands

## Abstract

Alzheimer’s disease (AD) is the leading cause of dementia worldwide. Even though the number of AD patients is rapidly growing, there is no effective treatment for this neurodegenerative disorder. At present, implementation of effective treatment approaches for AD is vital to meet clinical needs. In AD research, priorities concern the development of disease-modifying therapeutic agents to be used in the early phases of AD and the optimization of the symptomatic treatments predominantly dedicated to the more advanced AD stages. Until now, available therapeutic agents for AD treatment only provide symptomatic treatment. Since AD pathogenesis is multifactorial, use of a multimodal therapeutic intervention addressing several molecular targets of AD-related pathological processes seems to be the most practical approach to modify the course of AD progression. It has been demonstrated through numerous studies, that the clinical efficacy of combination therapy (CT) is higher than that of monotherapy. In case of AD, CT is more effective, mostly when started early, at slowing the rate of cognitive impairment. In this review, we have covered the major studies regarding CT to combat AD pathogenesis. Moreover, we have also highlighted the safety, tolerability, and efficacy of CT in the treatment of AD.

## 1. Introduction

Alzheimer’s disease (AD) is the leading cause of dementia and a complex chronic neurodegenerative disease [1,2]. The major risk factors of this multifactorial disease include apolipoprotein E *ɛ*4 (*APOE* ɛ*4*) genotype, family history, age, traumatic brain injury, hypercholesterolemia, obesity, hypertension, diabetes, and low education level [3,4]. The most vital causal factors for AD development are the presence of mutations in the genes encoding the amyloid precursor protein (*APP*), presenilin 1 (*PSEN1*), and presenilin 2 (*PSEN2*) [5,6]. Usually, at an early age (i.e., 30 to 50 years), around 50% of carriers of such mutations develop AD type dementia [7]. AD neuropathology includes synaptic dysfunction and neuronal loss in multiple brain areas; among those, the areas involved in cognition are mostly affected [8,9,10]. Indeed, the major AD hallmark includes the accumulation of Aβ as senile plaques and aggregating hyperphosphorylated tau-mediated neurofibrillary tangles (NFTs) [11,12]. Worldwide, about 50 million people are suffering from dementia, including AD. Moreover, by 2050, this aforesaid number is estimated to double [13,14].

Although the number of AD affected people is rapidly growing in the United States, there are currently only five approved treatment options that can be used to provide symptomatic treatments for AD [15]. In this regard, memantine (N-methyl-D-aspartate receptor (NMDAR) antagonist), constitutes the most recent treatment option which was approved more than 10 years ago [16]. On the other hand, four out of five of the standard treatments including memantine (NMDAR antagonist), rivastigmine, galantamine, and donepezil (cholinesterase inhibitors (ChEIs)) are licensed in the European Union [17,18,19]. The fifth treatment option is basically a combination of memantine and donepezil and this CT (i.e., Namzaric^®^) was approved in 2014 to treat individuals with moderate to severe AD, who are stabilized on donepezil and memantine therapy [20]. It involves the combination of two proven therapeutic agents (i.e., donepezil and memantine) in a fixed-dose combination product, providing the most effective way to start combination therapy (CT) in individuals with AD. Therefore, researchers are paying more attention to the multi-target-directed ligands (MTDLs) approach in order to develop hybrid molecules that simultaneously regulate multiple biological targets [21]. 

Memoquin is a novel drug, which has been developed as a potential anti-AD candidate because of its MTDL design approaches [22]. Moreover, MTDLs are formulated by the molecular hybridization of various pharmacophore subunits, from recognized biologically active molecules, which work as diverse ligands and which affect diverse biological targets [21]. Since AD is a multifactorial disorder, the combination of therapeutic agents may thus prove more effective as compared to single-agent therapy. In this article, we have critically reviewed the promising therapeutic options of CT for AD treatment. 

## 2. Widely Studied Combination Therapies for Alzheimer’s Disease 

Until now, the most widely studied combination drug therapy for AD treatment is the concomitant use of memantine and ChEIs. Furthermore, this treatment has proven clinical efficacy in AD treatment [23,24]. The effects of this CT in AD have also been assessed in long-term observational studies, open-label trials, and randomized controlled trials (RCTs). In AD, RCTs primarily evaluate drug efficacy, and these trials involve the determination of four main criteria including neuropsychiatric symptoms, functioning in activities of daily living (ADL), cognition, and global clinical outcomes. These criteria are regarded as demonstrative of clinical efficacy. The findings of these studies denote that CTs using memantine and ChEIs decrease the rate of functional and cognitive decline. Furthermore, as compared to no treatment or monotherapy with ChEIs, these CTs can reduce the emergence and the severity of neurobehavioral symptoms, for example, aggression/agitation, and delays nursing home admission [25,26,27,28,29,30,31,32,33,34,35,36], as shown in Table 1. It has also been demonstrated that combined therapies are more effective when started early [37].

### 2.1. Combination of Galantamine and Memantine

**Figure 1:**
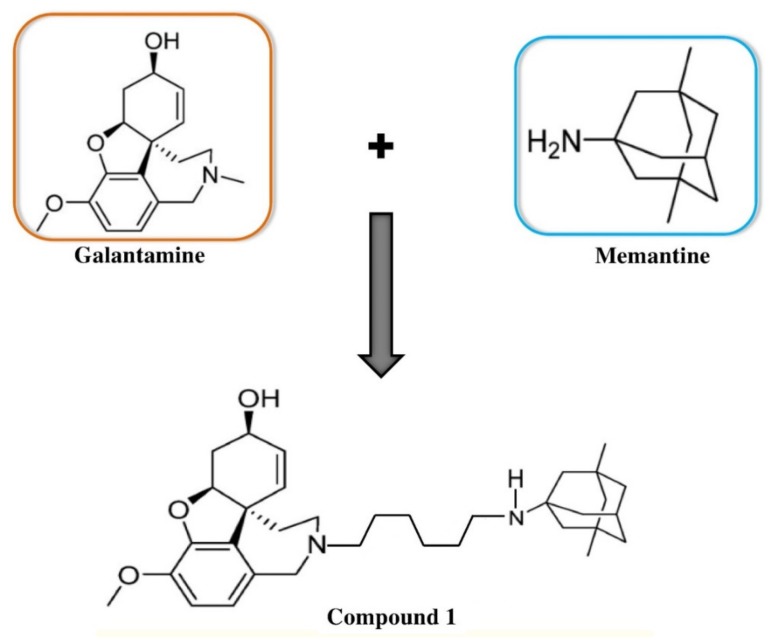
Chemical structure of one heterodimer of galantamine and memantine.

It has been suggested by biochemical evidence that there is a dysfunction in the activity of glutamatergic neurons in AD. Furthermore, their mutual dysfunction along with the cholinergic system is vital in Alzheimer’s pathology [40]. Therefore, CTs that target glutamatergic and cholinergic systems concurrently are considered as the present standard of care for individuals with AD [23]. It is known that NMDAR is accountable for learning and memory. Furthermore, glutamate and glycine are the agonists that need to simultaneously bind with the NMDAR for activating this receptor. It has been shown clinically that NMDAR overactivation (i.e., through an excess of glutamate) can cause an uncontrolled influx of Ca^2+^ into the neuron, which can eventually lead to excitotoxicity. On the other hand, along with its inhibitory effects on AChE, galantamine exhibits neuroprotective activities against glutamate toxicity through the activation of nicotinic ACh receptors [41]. Interestingly, smokers are less prone to Parkinson’s disease (PD) because of the induction of the nicotinic acetylcholine receptor (nAChR) in these individuals, which thus confers this neuroprotective effect [42]. Therefore, memantine and galantamine can act together in the same excitotoxic cascade to deliver a synergistic neuroprotective action [43]. The study by Lopes et al. [44] reinforced this hypothesis by demonstrating that galantamine’s neuroprotective action against NMDA-induced neurotoxicity in primary cortical neurons primarily involves nicotinic receptors. Furthermore, they also mentioned in their study that full neuroprotection might be achieved by combining subactive concentrations of memantine and galantamine. Additionally, their study also provides a new design strategy in the development of a MTDL with maximized efficiency [45]. A new series of hybrid compounds were designed by following this strategy that combined the pharmacological effects of memantine and galantamine [46]. On the other hand, following a dual-binding strategy, new hybrid compounds were designed by linking two drug molecules via a variable-length polymethylene linker (compound 1, Figure 1). In this series, compounds that carry a hexamethylene spacer are considered to display the most auspicious properties. A 6-methylene spacer offered the optimal distance to allow a concurrent contact with both the catalytic active site and the peripheral anionic binding site on AChE [46]. 

### 2.2. Combination of Memantine and Nitroglycerin

**Figure 2:**
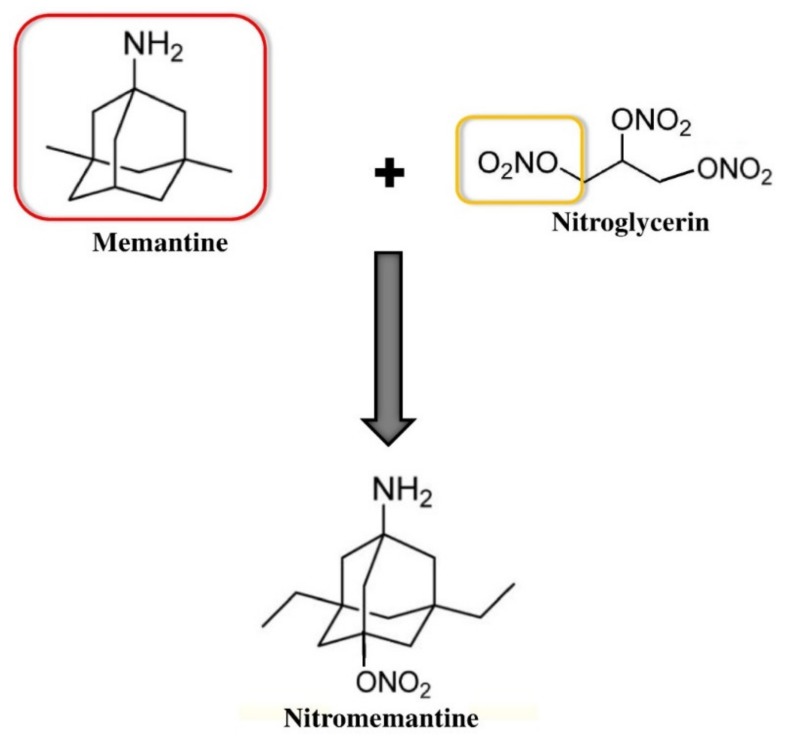
The linking of memantine and nitroglycerin leads to a new drug nitromemantine.

A second generation of memantine analogs (Figure 2) was developed to improve the efficacy of memantine and to provide it with a substantial disease-modifying effect [47]. An important pharmacophore (nitrooxy moiety, –ONO_2_) from nitroglycerin was linked with memantine to design these memantine analogs [48]. In a triple transgenic AD mouse model, the action of nitromemantine and memantine on extrasynaptic NMDARs was evaluated in vivo [49]. The triple transgenic AD mice showed impairments in cognition and synaptic function, which took place before extracellular Aβ deposition and tangles formation, but which was linked with intracellular Aβ immunoreactivity [49]. Nitromemantine exhibited greater effects, as compared to memantine, on elevating synaptic and dendritic density in 9-month-old triple transgenic AD mice. Furthermore, only the nitromemantine-treated group exhibited considerably better functions on the location-novelty recognition test. In addition to this, these studies revealed that nitromemantine was able to reverse the loss of brain connections and bring the number of synapses all the way back to normal within a few months of treatment in mouse AD models [50,51]. These aforesaid findings suggest that nitromemantine might have disease-modifying properties. Furthermore, nitromemantine did not show any negative effect on blood pressure [47,52]. Collectively, these promising results in animal models have prompted the entry of nitromemantine in clinical trials. These initiatives are indeed raising hope in the treatment of early and later-stages of AD.

### 2.3. Combination of Donepezil and Clioquinol 

**Figure 3:**
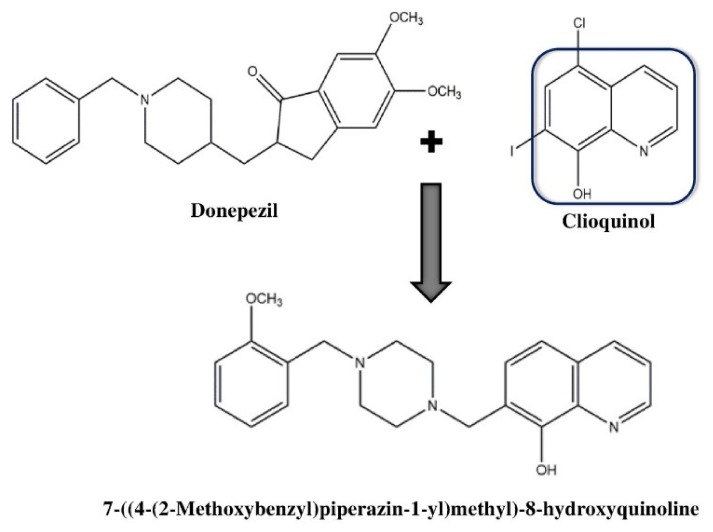
Chemical structure of one heterodimer of donepezil and clioquinol.

Generally, the linking approach produces molecules with poor pharmacokinetic properties and high molecular weight. Therefore, smaller hybrid molecules might prove beneficial as they possess better pharmacokinetic properties. Thus, a linking strategy was utilized to design a new series of hybrid molecules that combine the pharmacophore of donepezil with the metal chelator of clioquinol [53]. Amyloid plaques possess elevated concentrations of zinc and copper, which is also a common characteristic of inflammation [54]. In addition to this, amyloid toxicity can be mediated by the binding sites for both of these metal ions on Aβ oligomers [55]. Therefore, metal chelating therapy may be a good approach to offset AD progression. On the other hand, combined antioxidant, Cu(II)-complexing and cholinergic properties have been revealed in a series of tacrine-8-hydroxyquinoline hybrids [56]. New series of hybrid compounds were thus rationally designed based on these aforesaid observations through fusing the 8-hydroxyquinoline part with benzylpiperidines (i.e., as a donepezil pharmacophore) to attain inhibition of Aβ aggregation, free radical scavenging property, chelation of Zn(II) and Cu(II) and inhibition of ChE [57]. Substitution of the donepezil’s indanone core with the aromatic and planar 8-hydroxyquinoline preserve its affinity for the peripheral site of AChE without a rise in molecular weight. It was observed that most of the new hybrids obtained effectively suppressed the self-aggregation of Aβ and selectively targeted human butyrylcholinesterase (BuChE). Instead, the 7-((4-(2-methoxybenzyl)piperazin-1-yl)methyl)-8-hydroxyquinoline (Figure 3) exhibited good in vitro antioxidant properties, Zn(II)- and Cu(II)-chelating properties, acceptable toxicity, well-balanced anti-aggregating, and AChE properties as well as displaying positive permeation of blood-brain barrier dysfunction in parallel artificial membrane permeability assays.

### 2.4. Combination of Rivastigmine and Rasagiline

**Figure 4:**
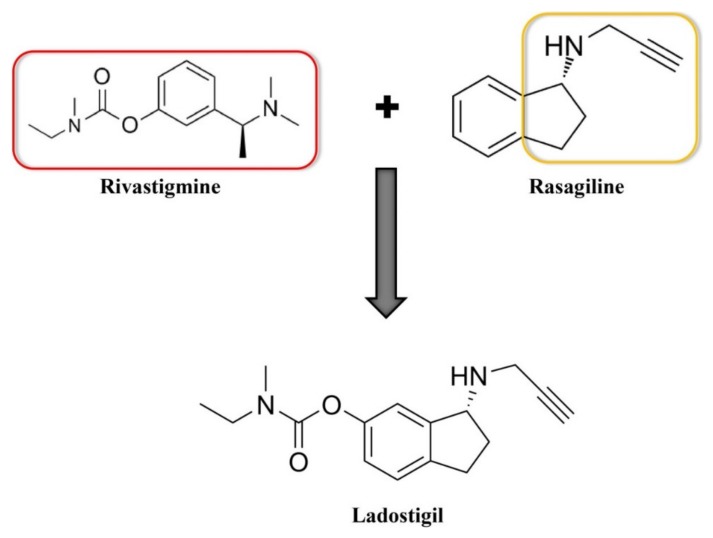
The linking of rivastigmine and rasagiline leads to a new drug ladostigil.

An example of successful molecular hybridization based on a drug combination strategy is the case of ladostigil (Figure 4), a neuroprotective agent currently undergoing clinical trials, that contains the characteristics of old drugs such as rasagiline (i.e., anti-monoamine oxidase (MAO)-B) and rivastigmine (i.e., anti-AChE). Ladostigil was studied in the treatment of AD, Lewy body dementia, and PD [58]. Moreover, this hybrid compound showed good reversible BuChE, AChE and irreversible MAO-B inhibition properties and protected impairments of motor function and memory in mice. Ladostigil (1 µM) also exhibited antioxidant properties via its direct scavenging activity on free radicals and also indirectly through the induction of the expression of cellular antioxidant enzymes [59]. It has been shown upon in vivo studies that ladostigil decreased the concentration of the holo-amyloid precursor protein (APP). In addition to this, ladostigil also increased the concentrations of protein kinase C (PKC)a, PKCe, and p-PKC in the mice hippocampus [60].

### 2.5. Combination of VK-28 and Propargylamine

**Figure 5:**
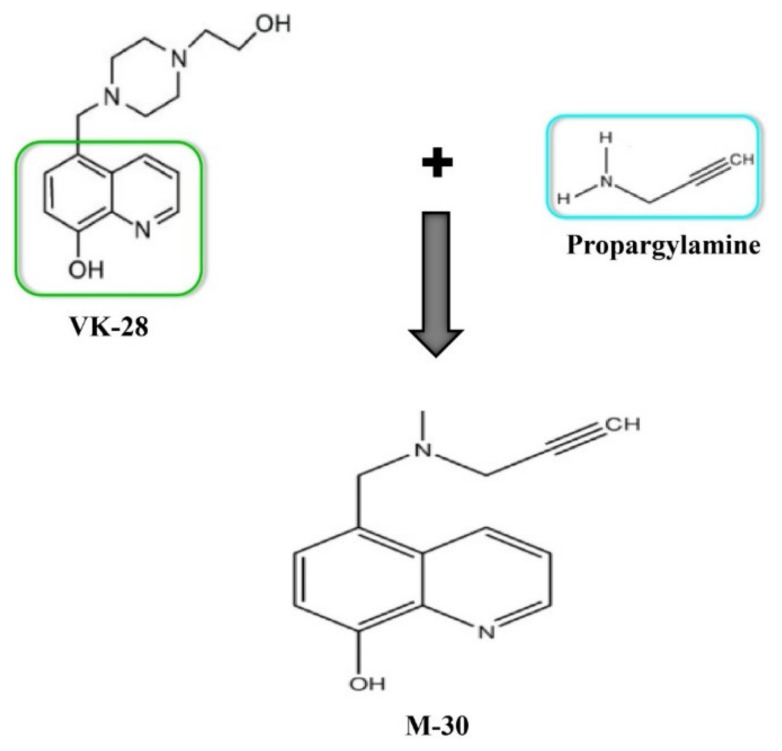
The linking of VK-28 and propargylamine leads to a new drug M-30.

M30 (Figure 5) was developed by combining the propargyl moiety of the anti-PD drug rasagiline with the skeleton of the neuroprotective iron chelator, VK-28. M30 possesses the vital pharmacophores (i.e., propargylamine moiety) from the Food and Drug Administration (FDA)-approved anti-PD rasagiline. The propargylamine moiety in the rasagiline’s structure plays a crucial role in the disease-modifying potential of rasagiline since it can mediate the interaction of this compound with various neuroprotective/neurorescue pathways [61]. PBT2 is a metal protein-attenuating compound (MPAC) of second-generation, which is now under phase II clinical trials for the treatment of AD. M30 possesses a metal protein attenuating/ionophore moiety similar to that of PBT2 [62]. MPACs typically show intermediate reversible affinity towards metals and have the capacity to reach certain intracellular compartments to target the harmful up-stream metal-protein reactions [63]. M30 displays a selective inhibition for MAO-A and MAO-B action in the brain, with a comparatively poor inhibition of these enzymes in the small intestine and liver in vivo [64]. 

M30 had a moderate affinity for metals (i.e., Zn, Fe, and Cu) like other MPACs, for example, clioquinol and PBT2. However, M30 showed a highly potent activity against the iron-stimulated mitochondrial membrane lipid peroxidation, which is comparable to the prototype iron chelator deferoxamine (DFO) [65]. The chronic treatment of aged mice with M30 (1 and 5 mg/kg; four times weekly for six months) in vivo exhibited a substantial positive effect on neuropsychiatry functions and age-related cognitive deficit [66]. Furthermore, in the treated mice, M30 considerably decreased the cerebral levels of Aβ and iron accumulation [67]. In this study, it was also revealed that the chronic administration of M30 reduces the cerebral pathology of Aβ and behavioral impairments in an APP/PS1 transgenic mouse model of AD [67].

## 3. Other Combination Approaches for Alzheimer’s Disease 

Although numerous efforts have been made, there is still no effective agent that can delay or stop the progression of AD. Multiple drugs including different combination treatment approaches focusing on the interference of the main pathological processes of AD are now under investigation. In Table 2, we have presented the available clinical data regarding these combination treatment options. 

### 3.1. Combination of PBT2 and ChEIs

Chronic exposure to various metals is associated with AD [105,106]. PBT2 (i.e., a copper/zinc ionophore) is a metal-chelating agent that is used to decrease the toxicity of protein-metal complexes like those produced by Aβ oligomers with zinc and copper. Early phase II clinical trials have already been completed with PBT2. Interestingly, in individuals with early AD, the combination of PBT2 and ChEIs (i.e., galantamine, donepezil or rivastigmine) enhanced executive functions in a dose-dependent manner and significantly decreased the levels of cerebrospinal fluid Aβ42 as compared to ChEIs alone [62,95]. Although these results are encouraging, further studies are required in this domain. 

### 3.2. Combination of Noradrenaline Reuptake Inhibitors and ChEIs/NMDAR Antagonists

Several studies have reported an intense noradrenergic dysfunction in AD [107,108]. Atomoxetine is an inhibitor of noradrenaline reuptake. In mild to moderately severe AD, it has been observed that CT, with atomoxetine and ChEIs (i.e., galantamine, 16–24 mg per day; donepezil, 5–10 mg per day; or rivastigmine, 6–12 mg per day) or an NMDAR antagonist (i.e., memantine 5–20 mg per day) was normally well tolerated by patients, but did not improve the monotherapy’s clinical efficacy in mild to moderately severe AD [94].

### 3.3. Combination of Anti-Tau Agents

Various treatment strategies mainly focus on tau pathology [11,109]. These strategies involve the use of drugs to inhibit tau phosphorylation, for example, inhibitors of the glycogen synthase kinase 3 (GSK-3), as well as antibodies or compounds that can reduce aggregation of tau. In individuals with moderate AD without previous psychosis or agitation, phase III clinical trials, including CTs with anti-tau agents, have studied the activity of flexible doses of valproate [88,89]. Chronic treatments with divalproex sodium neither slow functional and cognitive deficit nor delay the appearance of psychosis or agitation in patients with moderate AD [89]. Furthermore, patients also experienced noticeable toxic effects [89]. Moreover, individuals receiving valproate exhibited greater ventricular expansion, bigger loss in whole-brain and hippocampal volume and a faster deficit in the mini-mental state examination (MMSE) scores at month 12 [88,89].

### 3.4. Combination of Neurotrophic Agents 

In individuals with mild cognitive impairment (MCI) and AD, downregulation of the insulin-like growth factor-I (IGF-I), the brain-derived neurotrophic factor, the nerve growth factor, and IGF-I receptors has also been observed [110,111,112]. In fact, these decreases in neurotrophic signaling can cause the degeneration of basal forebrain cholinergic neurons and lead to an early event in the pathogenesis of AD [111]. On the other hand, neurotrophic changes are also linked with cognitive impairment, synaptic loss, decreased neural plasticity, apoptosis as well as Aβ- and tau-associated pathology [111,112]. Thus, mimetic peptides and drugs that can elevate brain neurotrophic signaling pathways are regarded as auspicious alternatives for AD treatment. No clinical efficacy has been observed with xaliproden (i.e., an agonist of the 5-hydroxytyiptamine 1A receptor) [90,91] and MK-677 (i.e., a growth hormone secretagogue) [92]. However, a combined treatment with cerebrolysin (i.e., a peptidergic drug) and donepezil [93] displayed some advantage over a monotherapy with donepezil in terms of enhancements in the global patient outcome, though more studies are required regarding this CT.

### 3.5. Combination of Antioxidative Factors

The imbalances of oxidants/antioxidants are strongly linked to several diseases including AD [113,114,115,116]. Oxidative damages constitute one of the pathological hallmarks of AD [117,118,119]. In several combination trials, vitamin E [98], group B vitamins [99] and omega-3 fatty acids [100,101] were ineffective to treat AD. 

### 3.6. Combination of Anti-inflammatory Drugs

Inflammation is intensely linked with several pathological conditions [120,121]. Elevated inflammatory signals are common in the pathogenesis of AD [122]. Unfortunately, all the phase III studies on CTs with anti-inflammatory agents were unsuccessful to exert clinical efficacy. In the combined AD treatment, ibuprofen [102] and celecoxib [103], rofecoxib and naproxen [104] did not exert any advantageous effects as compared to the placebo. 

### 3.7. Combination of Antidiabetic Drugs 

Insulin resistance plays a momentous role in the pathophysiological link between diabetes mellitus and AD [123]. Diabetes mellitus is closely related to cognitive impairment and AD [124]. In one study, patients with AD and type 2 diabetes mellitus have been treated with either oral antidiabetic drugs or a combination of insulin with other diabetes medications [96]. It has been observed that add-on therapy with insulin markedly decreased the functional and cognitive decline as compared to regular therapy without insulin in individuals with type 2 diabetes and mild-to-moderate AD [96]. 

## 4. Potential Benefits of Combination Therapies 

Monotherapy displays multiple limitations in the AD treatment including safety, efficacy, and disease modification. In addition to this, it is unlikely that a single agent targeting a single molecular target will cause sufficient alterations in AD pathophysiology to modify the progression of the disease. Furthermore, as single drug treatment is typically more effective at high doses, while as a result, it is likely to generate more severe side effects at these same doses. This is particularly applicable in the case of ChEIs. Their tolerability declines while efficacy elevates in a dose-dependent manner. In the framework of an effective pharmacological approach, treatment with a combination of drugs possessing different mechanisms of action may be more beneficial over monotherapy [23,24,125,126,127,128]. Moreover, CT might increase drug efficacy by stimulating synergistic or additive actions. CT may also improve tolerability and safety, thus allowing the use of lower doses [129]. Additional benefits of CT include other neuroprotective actions, extending the symptomatic benefits and eventually delaying the progression of the disease. 

### 4.1. Clinical Efficacy 

As stated earlier, it has been demonstrated through clinical studies that CT with memantine and a ChEI shows a higher efficacy in individuals with moderate-to-severe AD compared to monotherapy [35,38,39,130]. However, this greater efficacy was not confirmed in individuals with mild-to-moderate AD in RCTs of 24 weeks [32]. Nevertheless, observational studies in individuals with probable AD provided further evidence regarding CT’s long-term effectiveness in delaying nursing home admission of individuals across multiple disease stages [31] as well as in decreasing dependence level and cognitive impairment [25]. 

The efficacy of CT with memantine and donepezil has also been demonstrated upon an important RCT (i.e., the MEM-MD-02 study) in individuals with moderate-to-severe AD [35]. As compared to monotherapy, CT was linked with a markedly higher completion rate. Recently, in RCTs involving individuals with moderate-to-severe AD, noteworthy benefits of the CT with a memantine extended-release (28 mg per day) versus monotherapy with ChEIs were described for both cognition as well as global function [38,39]. 

### 4.2. Cognitive Effects 

The beneficial effects of CT have been demonstrated by several studies with memantine and ChEIs on cognition in individuals with moderate-to-severe AD. Improvement in cognitive function has also been shown by the MEM-MD-02 study group [35]. It has been revealed through a post hoc analysis that there is a statistically significant difference between the placebo and memantine groups in terms of language, memory, and praxis domains of cognition [33]. In individuals with moderate-to-severe AD who receive stable doses of ChEIs, marked beneficial effects on cognition were observed with the therapy involving extended-release memantine [38,39]. In line with this finding, beneficial effects on cognition were also observed with rivastigmine and memantine CT in individuals with AD exhibiting no response to rivastigmine alone after failing on galantamine or donepezil treatments [29].

However, the results of this CT on the cognitive effects in individuals with mild-to-moderate AD were less consistent. Regarding cognitive performances, the MEM-MD-12 study revealed no noticeable advantage of CT over monotherapy [32]. Furthermore, CT including rivastigmine and memantine patch did not exhibit any enhanced efficacy over monotherapy with rivastigmine patch in phase IV, multicenter, randomized, open-label clinical trial [27]. On the other hand, cognitive alterations after switching from donepezil to rivastigmine transdermal patches were also observed to be similar in individuals with mild-to-moderate AD without or with prior and concomitant treatment with memantine [28]. Nonetheless, individuals receiving previous memantine therapy had more severe functional and cognitive deficits and a markedly longer duration of the disease, thus findings of this trial are limited to the fact that the two experimental groups were not comparable. 

In a study aimed at determining the alterations in cognitive functions and brain volume, it has been revealed that introducing a treatment with memantine in individuals with mild-to-moderate AD (i.e., treated with AChEIs) enhanced their executive functions and language [36]. In another study, it was found that memantine treatment markedly enhanced the cognitive functions in individuals with mild-to-moderate AD who were on stable rivastigmine therapy [34]. Collectively, these findings suggest that CT has positive effects on executive functions, language, and attention in individuals with mild-to-moderate AD. 

### 4.3. Behavioral Effects 

Psychosis, agitation, and night-time disturbances are some of the neuropsychiatric symptoms that are usually linked with moderate-to-severe AD. Furthermore, these symptoms are very troublesome and one of the major causes of distress for care-givers. Therefore, any therapeutic interventions which decrease behavioral changes in AD patients, signify a relief for the caregivers as well as providing help in the maintenance of patients’ independence and their adhesion to treatment. As compared to monotherapy with donepezil, it has been observed that CT with memantine and donepezil leads to a marked decrease in the deterioration of neuropsychiatric symptoms in patients with moderate-to-severe AD [35]. 

The data resulting from further analysis of the trial have revealed that there are several factors acting in favor of the CT, including irritability/lability neuropsychiatric inventory items, eating/appetite, and agitation/aggression [27]. Interestingly, under CT patients who were agitated at baseline exhibited decreased scores of aggression/agitation but those who were free of agitation at baseline experienced a noteworthy delay in its occurrence. Furthermore, these results are in line with the findings of the pooled analyses of the behavioral effects of memantine in individuals with moderate-to-severe AD. Collectively, these findings reveal that memantine decreases the emergence or severity of aggression/agitation and the advancement of the global, functional and cognitive impairments that are shown by the placebo-treated agitated/psychotic individuals [130,131,132]. Nonetheless, in individuals with mild-to-moderate [26,32] or moderate-to-severe AD [29], other studies did not find marked effects of CT including memantine and ChEI on neuropsychiatric symptoms.

## 5. Safety and Tolerability of Combination Therapies

In general, as compared to monotherapy, the use of CT in AD is well tolerated without any major safety concerns. It has been concluded from a review that combined treatments including ChEIs and memantine were found to be well tolerated by patients [133]. However, dizziness and/or headaches were observed with the use of both of these agents. Furthermore, adverse events (AEs) specifically agitation and gastrointestinal were more prevalent in the case of the use of AChEIs, as compared to memantine. On the other hand, withdrawal cases in memantine-treated groups are comparable to placebo. Withdrawal cases were more common in AChEI-treated groups as compared to placebo. Collectively, there were fewer warnings, contraindications and drug-drug interactions with memantine as compared to AChEIs. Unfortunately, the use of AChEIs is contraindicated in individuals with cardiovascular complications due to their vagotonic effects. In the case of the use of AChEIs, precautions are needed in individuals with urinary outflow obstruction, seizures, obstructive pulmonary disease, asthma and risk of developing peptic ulcers. In contrast, the use of memantine is contraindicated in individuals with severe renal impairment and own hypersensitivity. Moreover, this agent should be used with caution in individuals with cardiovascular disease or a history of seizure [133]. Studies have also revealed that there are no pertinent differences between CT including memantine and ChEI versus monotherapy with ChEIs [25,31,32,38,39]. 

In patients with moderate-to-severe AD who received donepezil as monotherapy or in combination with memantine, a lower rate of treatment withdrawals due to AEs was observed in the combination group (7.4%) as compared to the donepezil monotherapy group (12.4%) [35]. On the other hand, the frequency of AEs was found to be comparable in individuals with mild-to-moderate AD treated with memantine and ChEIs and in those receiving ChEI monotherapy [32]. Over 5% of the CT-receiving individuals experienced a range of AEs including hypertension, fatigue, upper respiratory tract infection, confusion, diarrhea, gait abnormalities, depression, influenza-like symptoms, falls, accidental injury, dizziness, and agitation. 

## 6. Challenges of Combination Therapies in Alzheimer’s Disease

AD is a complex neurodegenerative disorder, thus treating AD patients still remains a challenge. Presently, the approved AD treatments are limited to memantine and ChEIs or the combination of these two agents. Most of the newly tested drugs failed in phase III trials due to their incapacity to meet the target efficacy, even though they exhibited promising results in their initial studies. The complex nature of AD is considered as one of the main reasons for the high failure rate of these new treatments [18]. Additionally, another reason for this high failure rate is our incomplete understanding of the numerous mechanisms involved in AD development and following neurodegeneration and the potential lack of efficacy of the available drugs. Since the combination of ChEIs and memantine have encountered less success in case of AD treatment, thus targeting multiple pathways to treat AD has better chances of success. Therefore, additional studies examining rational combinations of agents ought to be continued.

As add-ons to standard-of-care therapy, multiple current studies are currently exploring the effects of symptomatic agents or disease-modifying therapies. Nonetheless, a combination of two or more agents targeting different mechanisms and providing synergistic effects has better chances to treat AD. However, only a limited number of these agents are under clinical trials [134].

The use of ChEI-memantine CT in patients with mild-to-moderate AD has not been demonstrated conclusively. RCTs exhibited similar short-term performances for CT and monotherapy [32], while long-term observational analyses support the effectiveness of the CT to decrease the rate of cognitive decline and the level of dependence and denote that CT is more effective when started early and maintained for a long period [25,31,37,135]. Thus, long-duration RCTs are needed to confirm whether a CT applied in the early stages of AD can delay the progression of this disease. The potential long-term benefits of a CT should be more apparent after two years when the rate of deterioration is more evident [136]. 

CT might also diminish the doses used of the individual drugs with an eventual decrease in the costs and the side effects of the treatments. The design of adaptive and innovative clinical trials might be interesting for the potential development of therapeutic combinations over the disease progression, using one set of agents for preclinical AD, another for early-stage AD, and yet another for AD dementia. Collectively, challenges in AD treatment have guided the current therapeutic approaches toward the evaluation of new drugs as an add-on to standard-of-care and the repurpose of currently approved therapeutic agents which are indicated for other therapeutic conditions as well as the combination of agents that target different pathways [137]. 

Even though most of the add-on trials with potential disease-modifying drugs have failed so far, results in this area indicate that success may be further obtained, predominantly in case of neurotrophic agents and anti-amyloid strategies. Cerebrolysin, anti-Aβ vaccines and insulin may constitute beneficial agents which require additional studies in the future. Combinations of anti-amyloid and/or anti-tau interventions with neurotrophic agents have not yet been investigated but can lead to additive or synergistic effects. Further studies with drugs in prodromal or early AD stages may require long follow-up periods to prove their efficacy as the rates of progression of the disease and the clinical deterioration of patients are highly variable and rather slow [134]. 

## 7. Conclusions

AD is a complex disease, and there is a lack of effective single-target FDA-approved therapeutic agents. This situation has led to the development and the design of combined drug therapy. It is indeed difficult to combine several pharmacophores into a single molecule. However, this combination approach allows the simplification of the therapeutic regimen by the use of a single multi-target drug, which eventually ameliorates patient compliance. Presently, CT including ChEIs and memantine seems to constitute the best and effective treatment for individuals displaying moderate-to-severe AD. Additionally, CT exhibited better clinical efficacy as compared to monotherapy along with similar tolerability and safety. It is essential to carry out long-duration RCTs to establish whether CT delays disease progression in early AD stages. Other factors also need to be assessed in CT, such as its potential neuroprotective effects, cost-effectiveness, and a more exhaustive estimation of its potential benefits on the patients at the end-stage of AD. 

## Figures and Tables

**Table 1:** Clinical studies on combination therapy with cholinesterase inhibitors and memantine in Alzheimer’s disease.

Combination Therapy	Participants	Disease State	Duration	Study Design	Effects	References
ChEIs + memantine	47	Alzheimer’s disease (AD)	48 weeks	Single-arm, delayed-start exploratory study	No significant difference in the rates of total brain volume change between the two study periods. Treatment with memantine was also linked with superior performances on the executive functioning and reduction of right hippocampal atrophy.	[36]
Rivastigmine + memantine	172	Mild to moderate AD	24 weeks	Multicenter, randomized, open-label study	No significant differences in efficacy. No noticeable differences in safety and tolerability between the treatment groups.	[26]
Memantine + rivastigmine	176	Mild-to-moderate AD	25 weeks	Open-label, prospective, parallel-group study	No noticeable differences in tolerability between the treatment groups. No noticeable differences in cognition or global functioning.	[28]
ChEI + memantine	943	Probable AD	At least a 1-year follow-up	Real-world observational study on a long-term basis	Significantly extending time to nursing home admission.	[31]
Rivastigmine + memantine	90	Mild-to-moderate and moderate-to-severe AD	12 weeks	Open-label, pilot study	Enhancement of attention/executive function with secondary memory progress	[34]
ChEI + memantine	677	Moderate to severe AD	24 weeks	Multinational, randomized, double-blind, placebo-controlled, parallel-group trial	Significant improvements in severe impairment battery (SIB), clinician’s interview-based impression of change plus (CIBIC+) data, safe, and well-tolerated therapy.	[38,39]
ChEI + memantine	433	Mild to moderate AD	24 weeks	Randomized, double-blind, placebo-controlled trial	No statistically significant differences between the memantine- and placebo group on outcome measures.	[32]
Donepezil + memantine	404	Moderate to severe AD	24 weeks	Randomized, double-blind, placebo-controlled trial	As compared with monotherapy, combination showed significantly enhancement in SIB, less decline in AD Cooperative Study-Activities of Daily Living Inventory (ADCS-ADL) and improvement in CIBIC+ data.	[27,30,33,35]
ChEI + memantine	382	AD	4 years	Long-term real-world observational study	CT slows cognitive and functional impairment as compared to ChEI and no treatment.	[25]
Rivastigmine + memantine	202	Moderately severe AD	28 weeks	Open-label, multicentre study	Switching from donepezil or galantamine to rivastigmine may progress cognition and behavior. The addition of memantine may be useful.	[29]

**Table 2:** Clinical studies with agents targeting different pathophysiological mechanisms.

Therapeutic Agent	Target	Participants	Disease State	Duration	Combination Therapy Type	Study Design	Effects	References
Bapineuzumab	Amyloid pathology	234; 28; 210	Mild-to-moderate Alzheimer’s disease (AD)	78 weeks	Add-on	Phase II	No efficacy; Reduces cortical (11)C-PiB retention compared with both baseline and placebo	[68,69,70]
AN1792	Amyloid pathology	80; 159	Mild-to-moderate AD	4.6 years	Add-on	Phase IIA	Reduces neurite abnormality in the hippocampus; Decreases significantly functional decline	[50,71,72,73]
Intravenous immunoglobulins (Gammagard, Octagam, Flebogamma)	Amyloid pathology	58	Mild-to-moderate AD; Mild AD; AD	24 weeks; 6+9 months; 24 months	Add-on	Phase II; Open label dose-ranging study	Stabilized cognitive functions, reduced levels of Aβ in cerebrospinal fluid (CSF)	[51,74,75]
Semagacestat (LY450139)	Amyloid pathology	180; 51	AD; Mild-to-moderate AD	24 months; 14 weeks	Add-on	Phase III; Phase II	No efficacy; Decreases in plasma Aβ concentrations	[76,77,78]
Tramiprosate	Amyloid pathology	1052	Mild-to-moderate AD	78 weeks	Add-on	Randomized, double-blind, placebo-controlled, multi-centre trial	No clinical efficacy and reduced hippocampal atrophy	[79,80]
Etazolate (EHT0202)	Amyloid pathology	159	Mild-to-moderate AD	12 weeks	ChEIs	Phase IIA	Improves AD Cooperative Study-Activities of Daily Living Inventory (ADCS-ADL) data	[81]
Scyllo-inositol (ELND005)	Amyloid pathology	353	Mild-to-moderate AD	78 weeks	Add-on	Phase II	No significant primary clinical efficacy, and reduces CSF Aβ42 levels	[82]
Tarenflurbil	Amyloid pathology	1649; 210	Mild AD; Mild to moderate AD	18 months; 12+12 months	Add-on	Randomized controlled trial; Phase II	No clinical efficacy; Exerts a dose-related effect on measures of daily activities and global function	[83,84]
Rosiglitazone	Amyloid pathology	1496 + 1485	Mild-to-moderate AD	48 weeks		Two phase III studies	No clinical efficacy	[85]
Atorvastatin	Amyloid pathology	640	Mild-to-moderate AD	72 weeks	Add-on	Phase III	No clinical efficacy	[86]
Simvastatin	Amyloid pathology	406	Mild-to-moderate AD	18 months	Add-on	Phase III	No clinical efficacy	[87]
Divalproex sodium	Tau pathology	313	Moderate AD	24 months	Add-on	Phase III	No clinical efficacy	[88,89]
Xaliproden	Neurotrophic deficits	1455	Mild-to-moderate AD	18 months	Add-on	Phase III	No clinical efficacy	[90,91]
MK-677	Neurotrophic deficits	563	Mild-to-moderate AD	12 months	Add-on	Phase II	No clinical efficacy	[92]
Cerebrolysin	Neurotrophic deficits	197	Mild-to-moderate probable AD	28 weeks	Donepezil	Randomized, double-blind trial	Improves cognitive and global functions	[93]
Atomoxetine	Neurotransmitter Deficits	92	Mild-to-moderate AD	6 months	ChEIs	Phase II-III	No clinical efficacy	[94]
PBT2	Excitotoxicity	78	AD	12 weeks	ChEIs	Phase IIA	Improves cognition; Significant improvements in executive functions, and marked decrease in the CSF Aβ42 levels	[62,95]
Insulin	Metabolic alterations	104	Patients with mild-to-moderate AD and diabetes mellitus type-2	12 months	Add-on	Open-label	Reduces considerably cognitive decline	[96]
Hormone replacement therapy	Metabolic alterations	117	Menopausal women with AD	28 weeks	Rivastigmine	Randomized controlled trial	No significant effect	[97]
Vitamin E, Selegiline	Oxidative stress	341	Moderately severe AD	2 years	Combined versus monotherapy	Double-blind, placebo-controlled, randomized, multicenter trial	No superiority of combination to monotherapy	[98]
Folate/Vitamin B6/ Vitamin B12	Oxidative stress	409	Mild to moderate AD	18 months	Add-on	Multicenter, randomized, double-blind controlled trial	No effects on the primary cognitive measure	[99]
Docosahexaenoic acid	Oxidative stress	402	AD	18 months	Add-on	Randomized, double-blind, placebo-controlled trial	No clinical efficacy	[100]
Docosahexaenoic acid/Eicosapentaenoic acid	Oxidative stress	174	Mild to moderate AD	12 months	ChEIs	Randomized, double-blind, placebo-controlled trial	No clinical efficacy	[101]
Ibuprofen	Neuroinflammation	132	Mild to moderate AD	12 months	Add-on	Multicenter, randomized, double-blind, placebo-controlled, parallel group trial	No clinical efficacy	[102]
Celecoxib	Neuroinflammation	Conducted in 8 countries at 30 sites	Established mild-to-moderate AD	52 weeks	Add-on	Multicenter, randomized, double-blind, placebo-controlled, parallel-group trial	No clinical efficacy	[103]
Naproxen/Rofecoxib	Neuroinflammation	351	Mild-to-moderate AD	52 weeks	ChEIs	Multicenter, randomized, double-blind, placebo-controlled, parallel group trial	No clinical efficacy	[104]
